# Mesenchymal cell-derived extracellular vesicles ameliorate age-related deficits in working memory and *in vivo* MRI measures of white matter structure and function in rhesus monkeys

**DOI:** 10.1007/s11357-025-01992-0

**Published:** 2025-12-02

**Authors:** Evan C. Mackie, Chia-Hsin Cheng, Maya N. Alibrio, Christine Rutledge, Hongqi Xin, Michael Chopp, Ryan P. McCann, Douglas L. Rosene, Qiong Yang, Ella Zeldich, Maria Medalla, Bang-Bon Koo, Tara L. Moore

**Affiliations:** 1 Department of Anatomy & Neurobiology, Boston University Chobanian & Avedisian School of Medicine, 72 E Concord St, Room L1004, Boston, MA 02118 USA; 2 Center for Systems Neuroscience, Boston University, 610 Commonwealth Ave, Boston, MA 02215 USA; 3 Department of Neurology, Henry Ford Health, 2799 W Grand Blvd, Detroit, MI 48202 USA; 4 Graduate Program in Neuroscience, Boston University, 610 Commonwealth Ave, Boston, MA 02215 USA; 5 Department of Biostatistics, School of Public Health, Boston University, 715 Albany St, Boston, MA 02118 USA

**Keywords:** Normal aging, Rhesus monkey, Mesenchymal stromal cell-derived extracellular vesicles, Cognition, Diffusion MRI, Resting-state functional MRI

## Abstract

Aging humans and non-human primates both exhibit a similar pattern of cognitive decline beginning in middle age that is characterized by progressive impairments in rule learning, executive function, and working and recognition memory—functions often associated with dysfunction of prefrontal and medial temporal lobe regions. The heterogeneity and inter-subject variability in aging and age-related cognitive impairments present challenges for developing effective therapeutics and can be attributed to differing degrees of cortical white matter (WM) damage and alterations to local and long-range prefrontal and temporal networks. A promising therapeutic that has been shown to be efficacious in mitigating WM damage and improving cognitive function in rodent models is mesenchymal cell-derived extracellular vesicles (MSC-EVs). In the present study, late middle-aged rhesus monkeys were systemically administered monkey-derived MSC-EVs every 2 weeks for 18 months. We demonstrate that MSC-EV treatment improves spatial working memory and decreases the frequency of perseverative responses with largely no effects on recognition memory. These cognitive improvements were associated with increases in MRI diffusion measures of WM structural integrity over time as well as preservation of inter-network functional connectivity as measured by resting-state functional MRI. These findings suggest that MSC-EV treatment can slow or reverse age-related cognitive decline while strengthening WM integrity and improving functional connectivity in late middle-aged rhesus monkeys.

**Supplementary Information:**

The online version contains supplementary material available at https://doi.org/10.1007/s11357-025-01992-0.

## Introduction

Normal aging is characterized by deficits in the cognitive domains of learning, memory, and executive function [1–11]. Specifically, in humans, there are age-related changes in recall of information, speed of processing, visuospatial skills, and cognitive flexibility [12–17]. Similarly, in the aged rhesus monkey, a commonly used model of human aging, there are well-established changes in working memory, recognition memory, abstraction, set shifting, and perseveration [8, 9, 18–20]. These age-related declines are evident in performance on the Delayed Non-Matching to Sample Task (DNMS; recognition memory), the Delayed Recognition Span Task—Spatial (DRSTsp; working memory), and the Category Set Shifting Task (CSST; set-shifting, cognitive flexibility, and perseveration) in aged rhesus monkeys, with the earliest changes noted on the DRSTsp and CSST in middle age [8, 9, 18–25].

Compared to young monkeys, middle-aged and aged monkeys demonstrate shorter memory spans on the DRSTsp, reflecting deficits in spatial working memory [22, 23, 26–28]. Similarly, on the CSST, aged monkeys require more trials and make more errors when acquiring the first sorting principle (stimulus color) and on subsequent shift conditions, suggesting impairments in abstraction, set shifting, and cognitive flexibility [8, 9]. A particularly relevant deficit on both the CSST and DRSTsp is a strong perseverative response pattern shown by aged monkeys [8, 9, 27, 29], a behavior also commonly observed in aged humans [30–33]. Compared to the DRSTsp and CSST, changes in DNMS task performance are evident later in age. Monkeys of more advanced ages require more trials and commit more errors when acquiring the DNMS task than young and middle-aged monkeys, representing deficits in learning the task rule. Then on subsequent administrations of the task, they require more trials and make more errors in reacquiring task criterion and make more errors with increasing delays, suggesting an impairment in recognition memory [19, 20, 23, 34, 35]. These data support the heterogeneity of the progression, nature, and underlying regional pathology that may be associated with this cognitive decline with age.

While these age-related changes in cognition, referred to as “normal cognitive aging,” are well established in both humans and monkeys, the underlying drivers of these changes are not fully understood. It is known that cognitive impairments in aging rhesus monkeys are not a consequence of widespread cortical neuronal loss, but rather are thought to result, in large part, from myelin pathology in the frontal and medial temporal lobes [28, 36–39]. Alterations in white matter (WM) are also well documented in the normal-aged human brain. Extensive evidence in humans shows that cerebral WM integrity declines in healthy aging, as measured by diffusion tensor imaging, which is likely the result of changes in underlying myelin [40]. This is supported by a study with 100 individuals in a European consortium project that showed a relationship between age, cognitive performance, and degradation in WM integrity [41]. Furthermore, in aged rhesus monkeys, myelin integrity in the prefrontal cortex and frontal WM tracts declines, as shown by increases in myelin defects with electron microscopy and reduced fractional anisotropy with diffusion magnetic resonance imaging (MRI) [36, 42–44]. WM volume also decreases with age in monkeys, with the most pronounced loss occurring in the frontal lobe [28, 43, 45]. Importantly, age-related deterioration of WM does not occur in isolation but also disrupts neuronal health. Myelin and oligodendrocyte loss compromise axonal conduction and increase neuronal metabolic stress, thereby heightening neuronal vulnerability and contributing to neuronal network dysfunction [46–48]. Longitudinal imaging studies have also shown that declines in WM microstructure, such as increased diffusivity, often emerge before or alongside measurable gray matter atrophy in aging and disease, suggesting that WM deterioration may be an early driver of neuronal dysfunction [49–51]. These downstream effects highlight how WM pathology can propagate broader neurodegenerative changes that exacerbate cognitive aging.

The multifaceted nature and heterogeneity of normal cognitive aging present significant barriers to deciphering the precise cellular-molecular targets for the development of FDA-approved therapeutics to reverse age-related cognitive decline (ClinicalTrials.gov). Given that there is a potential correlation between age-related changes in WM integrity and cognition, therapeutics aimed at reversing WM pathology have begun to receive considerable attention. Extracellular vesicles (EVs) released by mesenchymal stromal cells (MSCs) carry bioactive proteins, microRNAs, and lipids and show promise as a therapeutic for reversing age-related changes in WM [52–61]. In rodent and primate models, MSC-EV treatment has been shown to enhance myelin plasticity by limiting damage to oligodendrocytes, stimulating remyelination, and promoting the differentiation of oligodendrocyte precursor cells into mature myelinating oligodendrocytes in human oligodendrocyte cultures [62–67]. Specifically, in experimental autoimmune encephalomyelitis (EAE) mouse models, MSC-EVs have been shown to reduce demyelination and improve functional outcomes [68–70]. Furthermore, our group has previously shown that MSC-EVs used in a rhesus monkey model of cortical injury ameliorate injury-related microglial inflammation and oligodendrocyte oxidative stress that enhance myelin plasticity [63, 71, 72]. However, it remains unknown whether MSC-EVs can ameliorate age-related progressive WM dystrophy in the aging primate brain and associated cognitive decline. To begin to fill this gap in our knowledge, the current study uses a rhesus monkey model of aging to investigate how chronic, systemic administration of MSC-EVs affects age-related cognitive decline and its underlying changes in in vivo WM structural integrity and functional connectivity. Our data provide evidence that in late middle-aged monkeys, long-term systemic treatment with MSC-EVs (every 2 weeks for 18 months) can slow or reverse age-related cognitive decline, particularly on a task of spatial working memory, while preserving WM integrity and functional network connectivity.

## Methods

### Subjects

Thirteen late middle-aged rhesus monkeys (17–24 years of age, equivalent to 51–72 human years) were used in this study (Table 1). All monkeys had known birth dates and complete health records and were obtained from National Primate Research Centers or private vendors. All monkeys received medical examinations before entering the study. In addition, explicit criteria were used to exclude monkeys with a history of splenectomy, thymectomy, exposure to radiation, organ transplantation, malnutrition, chronic illness including viral or parasitic infections, neurological diseases, or chronic drug administration. Each of the monkeys underwent an initial structural MRI scan to ensure there was no occult neurological damage. Results of the evaluations revealed that all monkeys were healthy at the time they were entered into the study.

**Table 1:** Biographical details of subjects used in this study

Subject	Sex	Group	DOB	Age at baseline testing	Age at retesting
AM380e	Male	Vehicle	5/22/96	24.4	26.7
AM384e	Male	Vehicle	11/10/03	18.2	20.2
AM405e	Male	Vehicle	3/22/98	24.7	26.7
AM414e	Female	Vehicle	11/1/03	19.2	21.1
AM415e	Female	Vehicle	5/1/03	19.5	21.4
SM071	Female	Vehicle	2/1/03	17.8	20.2
SM081e	Female	Vehicle	8/18/03	18.8	20.9
AM381e	Male	MSC-EVs	3/1/96	24.6	26.8
AM382e	Female	MSC-EVs	6/3/00	21.0	23.3
AM383e	Female	MSC-EVs	3/1/00	21.0	23.1
AM388e	Male	MSC-EVs	6/2/02	20.5	22.5
AM404e	Female	MSC-EVs	5/5/01	21.5	23.6
AM413e	Female	MSC-EVs	12/12/02	20.4	22.5

*DOB*, date of birth

While on study, monkeys were individually housed in colony rooms in the Boston University Animal Science Center, where they were in constant auditory and visual range of other monkeys. This facility is fully AAALAC accredited, and animal maintenance and research were conducted in accordance with the guidelines of the National Institutes of Health and the Institute of Laboratory Animal Resources Guide for the Care and Use of Laboratory Animals. All procedures were approved by the Boston University Institutional Animal Care and Use Committee. Diet consisted of Lab Diet Monkey Chow (#5038—Lab Diet Inc., St. Louis, MO, USA) supplemented by fresh produce with feeding taking place once per day, immediately following cognitive testing. During testing, small pieces of candy were used as rewards. Water was available continuously in the home cages, and monkeys were housed under a 12-h light/dark cycle with cycle changes occurring in a graded fashion over the course of an hour. Monkeys were checked daily by trained staff for health and well-being and were given a medical exam every 3 months by a clinical veterinarian.

### General study design

All thirteen monkeys received baseline MRI scans and cognitive testing consisting of initial Delayed Non-Matching to Sample (DNMS) acquisition, DNMS 2- and 10-min delays, and the Delayed Recognition Span Task—Spatial (DRSTsp). Monkeys were then randomly assigned to MSC-EV treatment (4 females and 2 males) or vehicle (4 females and 3 males) groups and began receiving intravenous (IV) infusions of either MSC-EVs or vehicle once every 2 weeks for 18 months (Table 1). Each monkey in the treatment group received a dose of 4 × 10^11^ MSC-EVs at each infusion, while monkeys in the vehicle group received an IV infusion of vehicle phosphate-buffered saline (PBS) that did not contain MSC-EVs. During the treatment period, MRI scans were repeated every 6 months. At the completion of the 18 months of treatment, monkeys were retested on cognitive tasks while continuing to receive infusions of MSC-EVs or vehicle every 2 weeks. All study personnel were blind to group assignments throughout the study. Due to limited availability of animals, we were unable to balance the groups for sex. Future studies will involve adding additional animals to fully assess sex differences in treatment efficacy.

### Baseline cognitive testing

**Fig. 1:**
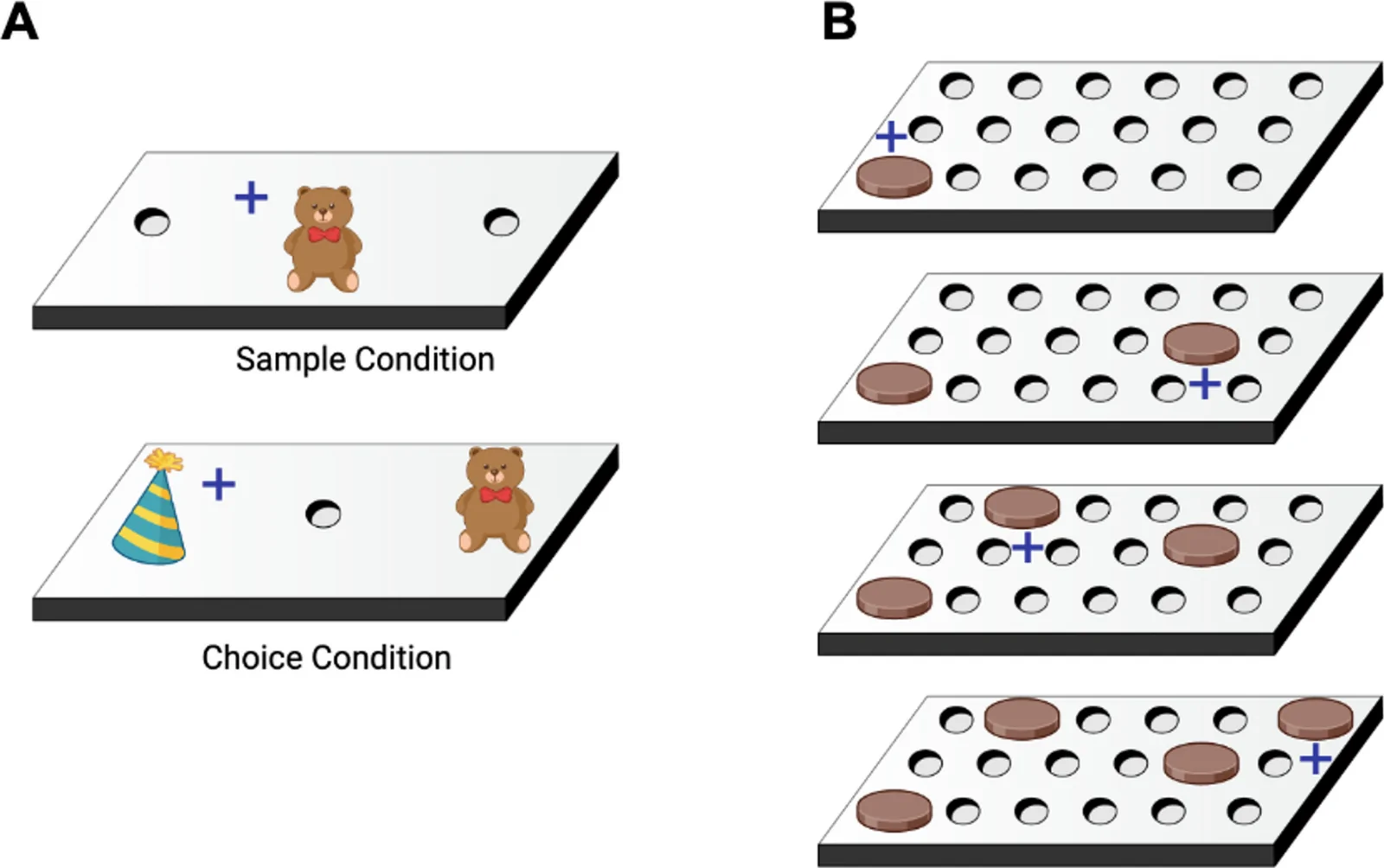
Schematic of the Delayed Non-Matching to Sample and Delayed Recognition Span Task—Spatial. **A** A schematic showing the sample and choice presentation during one trial of the Delayed Non-Matching to Sample (DNMS) task. In the sample condition, the monkey must displace the object presented over the center well to retrieve a food reward (blue cross). After a delay (10, 120, or 600 s), the monkey must now choose the new, unfamiliar object placed over a pseudorandomly chosen lateral well to obtain the food reward (blue cross). **B** A schematic showing sequential stimuli (brown disc) presentation within one trial of the Delayed Recognition Span Task—Spatial (DRSTsp). During each presentation the monkey must choose the disc in the new spatial location (blue cross). Each successive correct response trial was followed by the addition of a new disc in a novel location on the testing board and this continued until the monkey made an error (e.g.,, chose a previously chosen disc). With the occurrence of the first error, the trial was terminated and the number of discs on the testing board minus one was counted to determine the recognition span score for that trial (i.e., number of correct consecutive responses)

Prior to beginning treatments (vehicle or MSC-EV administration), all monkeys were initially familiarized with cognitive testing in a Wisconsin General Testing Apparatus (WGTA), where they were trained to displace a single gray plaque to obtain a reward. On each trial, the plaque was placed pseudorandomly over one of the three food wells, each 3.5 cm in diameter and 0.5 cm deep. Wells were spaced 11.5 cm apart in a single row across the middle of a testing board (the testing board that was also used for DNMS—see Fig. 1A). Small pieces of fruit or candy were used as rewards during testing. Monkeys were trained until they responded on 40 consecutive trials on each of two successive days. Then, the monkeys completed the DNMS task (acquisition and 2- and 10-min delays) and the DRSTsp.

### Initial acquisition—Delayed Non-Matching to Sample (DNMS)

The DNMS task is a benchmark recognition memory task that assesses the ability of subjects to identify a novel stimulus from a familiar stimulus following a 10 s delay interval. In the present study, all monkeys began the acquisition phase of the DNMS task in the WGTA. Using the same testing board as familiarization testing (Fig. 1A), each trial began with a sample object presented over the central baited food well. The monkey was permitted to displace the object and obtain the reward. The WGTA door was then lowered, and the original, now familiar, sample object was placed over an unbaited lateral well and a novel, unfamiliar object was placed over the other lateral well that was baited. Ten seconds after the original sample trial, the choice trial was begun, and the monkey was required to choose the unfamiliar, novel object to obtain the reward. Both testing objects were replaced after each trial so as not to reuse objects during the same testing period. Ten seconds later, the next trial was initiated with a new, novel sample object presented over the baited central well. Ten seconds later, another recognition trial was given using the second sample object and another new novel object. The position of the two objects on successive recognition trials was varied from the left to right lateral wells in a pre-determined pseudorandom order. A non-correction procedure was used and twenty trials per day were given until the monkey reached a learning criterion of 90 correct responses in 100 consecutive trials or a maximum of 2000 trials. Objects were drawn from a pool of 600 “junk” objects and paired so that in each daily session of 20 trials, 60 of the objects were used. Once all the initial pairings were used (30 days of 20 trials per day), the 600 objects were randomly recombined to produce new pairs so that the pairings presented continued to be new and unique on each trial.

### Delayed Non-Matching to Sample (DNMS) 2-min delays

Upon reaching criterion on the basic task, the 10 s delay between the presentation of the sample object and the recognition trial was increased to 120 s. Ten trials a day for 5 days (for a total of 50 trials) at the delay interval were administered with the monkey remaining in the testing apparatus during the delay interval.

### Delayed Non-Matching to Sample (DNMS) 10-min delays

Upon completion of the trials with the 2-min delay between the presentation of the sample object and the recognition trial, the delay was increased to 600 s. Five trials a day for 5 days (for a total of 25 trials) at the delay interval were administered with the monkey remaining in the testing apparatus during the delay interval.

### Delayed Recognition Span Task—Spatial (DRSTsp)

The testing board for the DRSTsp was similar to the DNMS board (Fig. 1A), but, instead of a single row of three wells, this board had three rows of six wells each (3.5 cm wide, 0.5 cm deep) and the wells were spaced 6 cm apart within a row. The rows were spaced 1.5 cm apart (Fig. 1B). For this task, fifteen identical plain brown discs (6 cm in diameter) were used as stimuli. During the first sequence of a trial, one disc was placed over one of the eighteen wells, which was baited with a food reward. The WGTA door was then raised, and the monkey was allowed to displace the disc to obtain the reward. The door was then lowered, the first disc was returned to its original position over the now unbaited well, and a second disc was placed on the board over a baited well in a different location. After 10 s, the door was once again raised, and the monkey was required to identify the new second disc in its novel spatial location to obtain the reward. Each successive correct response trial was followed by the addition of a new disc in a novel location on the testing board and this continued until the monkey made an error (i.e., chose a previously chosen disc). With the occurrence of the first error, the trial was terminated and the number of discs on the testing board minus one was counted to determine the recognition span score for that trial (i.e., number of correct consecutive responses). Five such trials were presented each day for 10 consecutive days (a total of 50 trials).

### MRI scanning

Each monkey underwent baseline structural MRI to ensure there was no occult neurological damage, and scans were repeated every 6 months during treatment and prior to euthanasia after completion of the study protocol. All scans were conducted on the 3 Tesla whole body wide bore Philips Ingenia Elition scanner (Center for Biomedical Imaging, Boston University Chobanian and Avedisian School of Medicine). Due to a scanner upgrade that occurred mid-study, we have focused on analyzing MRI data collected after the scanner upgrade to ensure consistency in data.

For each scan session, monkeys were placed into a custom-designed acrylic stereotaxic device that was created to allow for the imaging of monkeys using the 8-channel, phase-array Philips head coil. This ensures homogeneity of the MR field and even application of the signal from the radio frequency pulse (i.e., no drop-off in signal) along with access to the standard set of filters and image acquisition tools on the scanner. For scans, monkeys were initially sedated with ketamine (10 mg/kg IM) which was followed with additional doses, as needed.

The following scans were acquired in each MRI session: (1) A structural MRI scan was acquired using a T1-weighted 3D-turbo field echo (TFE) sequence that was fully optimized to provide a high signal-to-noise ratio (SNR) and high gray-white matter contrast at high resolution with the following parameters: Repetition time (TR)/Echo time (TE) = 7 ms/3 ms, flip angle = 8°, number of excitations/averages (NEX) = 8, voxel size = 0.7 × 0.7 × 0.7 mm^3^, sagittal plane acquisition. (2) Diffusion MRI (dMRI) was collected using a diffusion-weighted imaging spin-echo (DWIse) sequence modified based on the Adolescent Brain Cognitive Development (ABCD) multi-band (MB) diffusion sequence with the following parameters: TR/TE = 4200 ms/67 ms, flip angle = 90°, number of directions = 108, b-values = 500, 1000, 2000, 3000 m/s, voxel size = 1.875 × 1.875 × 2 mm^3^. (3) Resting-state functional MRI (rs-fMRI) was collected from a Fast Field Echo (FFE) single-shot echo planar imaging (EPI) sequence with the following parameters: TR/TE = 2000 ms/30 ms, flip angle = 52°, slice thickness = 2.34 mm, 30 slices, field of view (FOV) = 150 × 150 × 70.19 mm^3^, and 200 volumes.

### MRI processing and analysis

Prior studies from our group have utilized a combination of various diffusion and functional MRI techniques and advanced reconstruction methods to assess WM integrity, functional network connectivity, and structural–functional properties in the aging rhesus monkey brain. In the current study, we sought to expand on these findings by adopting a model-free diffusion reconstruction method, q-space sampling [73], to detect more detailed microstructural WM changes. Recent studies with resting-state fMRI have defined large-scale functional brain networks (e.g., default mode network, executive network, salience network) in the human brain by grouping brain regions that share similar functional properties into the same network with a lesser focus on anatomical organization and boundaries [74] which have been modified onto the rhesus monkey brain [75]. In this study, we focused on changes in WM integrity using dMRI, resting-state networks (RSNs) using rs-fMRI, as well as the relationship between MRI measures and cognitive performance outcomes to better understand the therapeutic role of MSC-EVs.

All raw MR images were converted and preprocessed using a modified in-house pipeline [76]. In this study, T1 and rs-fMRI data were processed using the AFNI (analysis of functional NeuroImages) macaque fMRI processing pipeline [77], which provided hierarchical cortical regions of interest (ROI) parcellation that can be further mapped into major RSNs by matching anatomical and neurological proximity between NHP and human brain functional networks [75]. ROI-based mean connectivity correlations were calculated for 5 major RSNs (limbic, default mode, salience, control, and executive networks) and 10 inter-network connections (i.e., averaged correlations between RSNs) were calculated from the pipeline [77]. A total of 15 functional network features were used for the analysis. Diffusion MRI processing included generalized q-space imaging (GQI) reconstruction from DSI Studio, a model-free method to evaluate microstructural diffusivity measured as the generalized fractional anisotropy (GFA) [73], which offers better estimation of WM crossing fibers, axonal integrity, and myelination [78]. B0 images were then registered to the standard NIMH Macaque Template (NMT) brain template [79]. A diffusion-tensor imaging (DTI)-based WM atlas containing 57 WM labels was then inverse transformed to the dMRI space to provide matching anatomical definitions in the reconstructed GFA maps [80].

### Mesenchymal stromal cell-derived extracellular vesicle (MSC-EV) preparation

EVs were isolated from bone marrow MSCs of a young monkey, as described previously [81]. Briefly, a young monkey (approximately 6 years old—equivalent to 18 human years) was sedated with ketamine (10 mg/kg IM), then anesthetized with sodium pentobarbital (15–25 mg/kg IV). Bone marrow was extracted from the iliac crest and shipped on wet ice with same day delivery to Henry Ford Health in Detroit. Upon arrival, marrow was spun at 4000 × g for 15-min to separate cells. The buffy coat was discarded, and remaining cells were washed in culture medium. Cells were then plated in a T75 flask using media containing 20% FBS and alpha-MEM, grown to confluence, and passaged as necessary. To grow enough cells for EV isolation, 10 × 10^6^ cells were seeded into a Quantum Incubator (Terumo BCT, Lakewood, CO, USA) and grown in alpha-MEM with 10% EV-depleted FBS (Systems Biosciences, Palo Alto, CA, USA). To harvest EVs, media was collected every other day for four days, then every day for two days. As described in Zhang et al., 2015 [82], media was centrifuged in multiple stages at 250 × g for 5 min, then 3000 × g for 30 min, then filtered and centrifuged a final time at 100,000 × g for 2 h to pellet EVs. The pellet was then resuspended in a small volume of PBS. EV concentrations and particle sizes were measured using a qNano (Izon, Cambridge, MA, USA), then diluted to 4 × 10^11^ particles for each monkey in 10 mL of PBS. EVs had an average diameter of 111 nm in size and had confirmed expression of CD9, CD81, and CD63 by Western blot, per MISEV guidelines (data not shown) [83]. The dose was chosen based on previous dosing studies conducted in rats [82, 84] and is consistent with the MSC-EV dose used in our prior studies on recovery from cortical injury [63, 71, 81, 85]. Following resuspension, MSC-EV or PBS vehicle vials were individually labeled with subject identifiers, stored at −80 °C, and shipped to Boston University. The day prior to intravenous administration in monkeys, treatment vials were allowed to thaw overnight at 4 °C.

### MSC-EV administration

Following the completion of baseline testing, the monkeys were randomly assigned to either the MSC-EV treatment or vehicle (control) groups and began receiving IV dosing of MSC-EVs or vehicle once every 2 weeks. For each administration, monkeys were sedated with ketamine (10 mg/kg IM) and then an IV catheter was inserted into a branch of the saphenous vein. An infusion set was then used to administer 10 ml of MSC-EVs (4 × 10^11^ particles in 10 mL of PBS) or vehicle. Dosing was continued for 18 months.

At the end of the 18-month dosing period, the monkeys began retesting on the DNMS task and the DRSTsp while continuing to receive the biweekly doses of MSC-EVs or vehicle. Reacquisition of the DNMS task was administered as described above and monkeys were retested until reaching a criterion level of 90 correct responses in 100 consecutive trials. The DNMS delays and DRSTsp were then administered as described above.

### Data analyses and statistics

#### Analyses of cognitive data

Analyses of cognitive data were primarily conducted using GraphPad Prism (Version 10.2.3 for macOS, GraphPad Software, Boston, MA, USA). Standard two-way analyses of variance (ANOVAs) were used to evaluate the main and interactive effects of treatment group and sex. For comparisons across baseline and retesting sessions or across trial blocks, two-way repeated measures ANOVAs were applied, with time (or trial block) treated as the within-subjects factor and treatment group as the between-subjects factor. For single time-point comparisons (baseline or retesting only), standard two-way ANOVAs were performed. All statistical tests were performed with an alpha level of 0.05 for statistical significance. Data are presented as group means ± standard error of the mean (SEM).

To complement these Prism-based analyses, confirmatory linear mixed-effects models were run in IBM SPSS Statistics (Version 29.0; IBM Corp., Armonk, NY, USA, 2023). Mixed models were applied only to outcomes that showed significant effects in the ANOVA framework. This approach allowed us to account for repeated measures within subjects, inter-individual variability, and potential baseline imbalances while providing robust parameter estimates using HC3 robust standard errors. Fixed effects included treatment group, sex, age, and time (where applicable), with subject entered as a random effect. Model outputs are reported as parameter estimates with robust standard errors, test statistics, *p*-values, and 95% confidence intervals.

To assess DNMS performance at both baseline testing and retesting, the total number of trials and errors to reach criterion during baseline acquisition and reacquisition after 18 months were calculated for each monkey (Table 2). To determine if there were trends in the change in performance across testing days during reacquisition, the number of errors made by each monkey on each day of testing before reaching criterion (excluding the last 100 trials representing the reacquisition) was also determined (Supplementary Fig. 1). DNMS performance with 2- and 10-min delays was quantified by calculating, for each monkey, the percentage of correct trials over the five days of testing for each delay at baseline and retesting.

**Table 2:** Subject cognitive performance data on the Delayed Non-Matching to Sample (DNMS) task

Subject	Sex	Group	Baseline				Retesting							
			DNMS total trials	DNMS total errors	DNMS 2-min delays % correct	DNMS 10-min delays % correct	DNMS Re-acq total trials	DNMS Re-acq total errors	DNMS trials normalized to baseline	DNMS errors normalized to baseline	DNMS 2-min delays % correct	DNMS 2-min normalized to baseline	DNMS 10-min delays % correct	DNMS 10-min normalized to baseline
AM380e	Male	Vehicle	923	214	74%	60%	360	93	−61.0	−56.50	56%	−24.32	64%	6.67
AM384e	Male	Vehicle	800	242	74%	96%	280	43	−65.0	−82.20	88%	18.92	84%	−12.50
AM405e	Male	Vehicle	775	250	90%	72%	180	39	−76.8	−84.40	84%	−6.67	56%	−22.22
AM414e	Female	Vehicle	460	139	76%	44%	40	10	−91.3	−92.80	76%	0.00	64%	45.45
AM415e	Female	Vehicle	835	192	80%	80%	320	79	−61.7	−58.90	60%	−25.00	56%	−30.00
SM071	Female	Vehicle	930	261	81%	68%	0	0	−100.0	−100.00	82%	1.23	64%	−5.88
SM081e	Female	Vehicle	606	140	82%	84%	265	51	−56.3	−63.60	76%	−7.32	56%	−33.33
		**Mean**	**761.29**	**205.43**	**79.57%**	**72.00%**	**206.43**	**45.00**	**−73.15**	−**76.91**	**74.57%**	−**6.17**	**63.43%**	−**7.40**
		**SE**	**64.85**	**19.12**	**2.14**	**6.41**	**52.63**	**12.71**	**6.35**	**6.53**	**4.59**	**5.79**	**3.75**	**10.26**
AM381e	Male	MSC-EVs	873	216	64%	72%	320	75	−63.3	−65.30	80%	25.00	72%	0.00
AM382e	Female	MSC-EVs	760	208	86%	64%	260	79	−65.8	−62.00	92%	6.98	64%	0.00
AM383e	Female	MSC-EVs	336	78	68%	72%	320	68	−4.8	−12.80	68%	0.00	60%	−16.67
AM388e	Male	MSC-EVs	951	279	70%	80%	20	3	−97.9	−98.90	80%	14.29	64%	−20.00
AM404e	Female	MSC-EVs	520	149	66%	86%	60	13	−88.5	−91.30	80%	21.21	84%	−2.33
AM413e	Female	MSC-EVs	670	247	72%	68%	80	23	−88.1	−90.70	72%	0.00	60%	−11.76
		**Mean**	**685.00**	**196.17**	**71.00%**	**73.67%**	**176.67**	**43.50**	−**68.07**	−**70.17**	**78.67%**	**11.25**	**67.33%**	−**8.46**
		**SE**	**93.27**	**29.53**	**3.21**	**3.28**	**56.43**	**13.96**	**13.83**	**13.00**	**3.37**	**4.35**	**3.78**	**3.62**

To assess DRSTsp performance at both baseline testing and retesting, the mean total span achieved by each monkey was determined across all 50 baseline or retest trials. The number of perseverative errors made by each monkey was also determined across all 50 trials at each timepoint (Table 3). A linear analysis of change in task performance was conducted to assess the direction of the change in each group for a subset of outcome measures (DRSTsp total span and perseverative errors). A vertical scatter plot of baseline and retesting data from individual subjects was plotted, and a linear regression line was fit through the data from each subject. The linear slope was calculated for each group between baseline and retesting scores (Supplementary Fig. 2). To determine if there were trends in the change in performance across testing days on the DRSTsp, the span of each of the 50 trials was determined for monkeys in both the vehicle and MSC-EV groups at retesting (Supplementary Fig. 3). Spans were then averaged across blocks of 5 trials (one testing day) for each monkey.

**Table 3:** Subject cognitive performance data on the Delayed Recognition Span Task—Spatial (DRSTsp)

Subject	Sex	Group	Baseline		Retesting			
			DRSTsp total span	DRSTsp PEs	DRSTsp total span	DRSTsp PEs	DRSTsp total span normalized to baseline	DRSTsp PEs normalized to baseline
AM380e	Male	Vehicle	2.08	22	1.76	32	−15.38	45.45
AM384e	Male	Vehicle	2.20	27	2.24	29	1.82	7.41
AM405e	Male	Vehicle	2.10	23	2.4	23	14.29	0.00
AM414e	Female	Vehicle	1.86	22	1.76	22	−5.38	0.00
AM415e	Female	Vehicle	2.34	17	1.94	25	−17.09	47.06
SM071	Female	Vehicle	2.54	21	2.2	29	−13.39	38.10
SM081e	Female	Vehicle	2.52	26	2.14	19	−15.08	−26.92
		**Mean**	**2.23**	**22.57**	**2.06**	**25.57**	−**7.17**	**15.87**
		**SE**	**0.09**	**1.25**	**0.09**	**1.74**	**4.40**	**10.63**
AM381e	Male	MSC-EVs	2.18	32	2.64	23	21.10	−28.13
AM382e	Female	MSC-EVs	1.90	21	2.38	16	25.26	−23.81
AM383e	Female	MSC-EVs	2.32	29	2.64	22	13.79	−24.14
AM388e	Male	MSC-EVs	2.06	23	2.8	22	35.92	−4.35
AM404e	Female	MSC-EVs	2.06	19	3.06	20	48.54	5.26
AM413e	Female	MSC-EVs	2.08	18	2.86	21	37.50	16.67
		**Mean**	**2.10**	**23.67**	**2.73**	**20.67**	**30.35**	**−9.75**
		**SE**	**0.06**	**2.30**	**0.09**	**1.02**	**5.16**	**7.52**

*DRSTsp*, Delayed Recognition Span Task—Spatial; *PEs*, perseverative errors

To assess changes in cognitive performance over time, baseline-normalized scores for the DNMS task (number of trials and errors, and percentage of correct trials at 2- and 10-min delays) and DRSTsp (total span and perseverative errors) outcome measures between baseline testing and retesting for each animal were calculated (Tables 2 and 3). For each measure and subject, the normalized scores were calculated with the following formula:

Normalized score = $\left[100\times \left(\frac{\left(\text{baseline score}-\text{restest score}\right)}{\text{baseline score}}\right)\times -1\right]$. This analysis provides a value to represent the percent difference between performance at baseline and retesting for each outcome measure.

#### MRI data analysis

MRI group differences between MSC-EV-treated and vehicle monkeys at each available time point were assessed with two-way ANOVAs adjusting for treatment, sex, and the interaction between treatment and sex (MATLAB, R2024a, The MathWorks Inc. Natick, MA, USA). Partial correlation analyses adjusting for age and sex as variables were performed between MRI and cognitive testing measures. Post-hoc corrections for multiple comparisons were reported by the false discovery rate (FDR) *p*-value [86]. Pearson correlation coefficient (r) or *F*-statistics is reported with the raw and FDR-corrected *p*-value for significant comparisons (MATLAB, R2024a, The MathWorks Inc. Natick, MA, USA). All statistical tests were performed with an alpha level of 0.05 for statistical significance.

To assess longitudinal treatment effects over time, the change in dMRI measures was normalized to baseline with the following formula:

White matter dMRI–normalized rate change = $\frac{(\frac{\text{terminal dMRI}-\text{baseline dMRI}}{\text{baseline dMRI}})}{{\mathrm{age}}_{\text{terminal }}-{\mathrm{age}}_{\mathrm{baseline}}}$.

Group differences in the normalized dMRI measures between treated and vehicle monkeys were assessed with two-way ANOVAs as described above. Additionally, correlation analyses adjusting for age and sex as variables were performed between the baseline-normalized dMRI measure and the baseline-normalized DRSTsp scores.

## Results

### Baseline Cognitive Testing

#### Delayed Non-Matching to Sample—Initial Acquisition

Initial acquisition on the DNMS task was used to measure learning efficiency, quantified as the number of trials and errors required to reach criterion performance (Table 2). Two-way ANOVA revealed no significant effect of group on DNMS outcome measures at baseline. There was no significant main effect of group and no sex by group interaction effects on the number of trials (group, [*F*(1, 9) = 0.0853, *p* = 0.777]; sex × group, [*F*(1, 9) = 1.224, *p* = 0.2972]) or errors (group, [*F*(1, 9) = 2.593e-5, *p* = 0.996]; sex × group, [*F*(1, 9) = 1.41, *p* = 0.715]). However, female monkeys required significantly fewer trials to criterion than males (639.6 ± 101.6 vs. 864.4 ± 133.2; [*F*(1,9) = 5.706, *p* = 0.0406]; two-way ANOVA).

To account for the possibility that this observed sex effect might be attributable to age differences between males and females (Table 1), we included age as a covariate in mixed-model analyses. These results confirmed that age was not a significant predictor of DNMS acquisition performance (mixed model, [*F*(1,9) = 0.150, *p* = 0.712]), and the sex effect persisted after adjustment, although at trend-level significance (mixed model: [*F*(1,9) = 5.013, *p* = 0.052]). This indicates that the female advantage in acquisition trials was not explained by differences in age between males and females.

#### Delayed Non-Matching to Sample—Delays

Performance on DNMS delays was assessed as the percentage of correct trials at 2- and 10-min delays, reflecting short- and longer-term recognition memory (Table 2). At baseline, monkeys assigned to the vehicle group performed significantly better on 2-min delays (80 ± 2%) than monkeys assigned to MSC-EV group (71 ± 3%; [*F*(1,9) = 5.421, *p* = 0.0449]; two-way ANOVA). However, there was no significant main effect of group on 10-min delays ([*F*(1, 9) = 0.04238, *p* = 0.8415]). Furthermore, there was no significant main effect of sex, or sex by group interaction effects on the percentage of correct trials at both 2-min (sex, [*F*(1, 9) = 0.612, *p* = 0.454]; sex × group [*F*(1, 9) = 0.464, *p* = 0.513]) or 10-min delays (sex, [*F*(1, 9) = 0.381, *p* = 0.552]; sex × group [*F*(1, 9) = 0.04, *p* = 0.842]).

#### Delayed Recognition Span Task—Spatial—Total Span

**Fig. 2:**
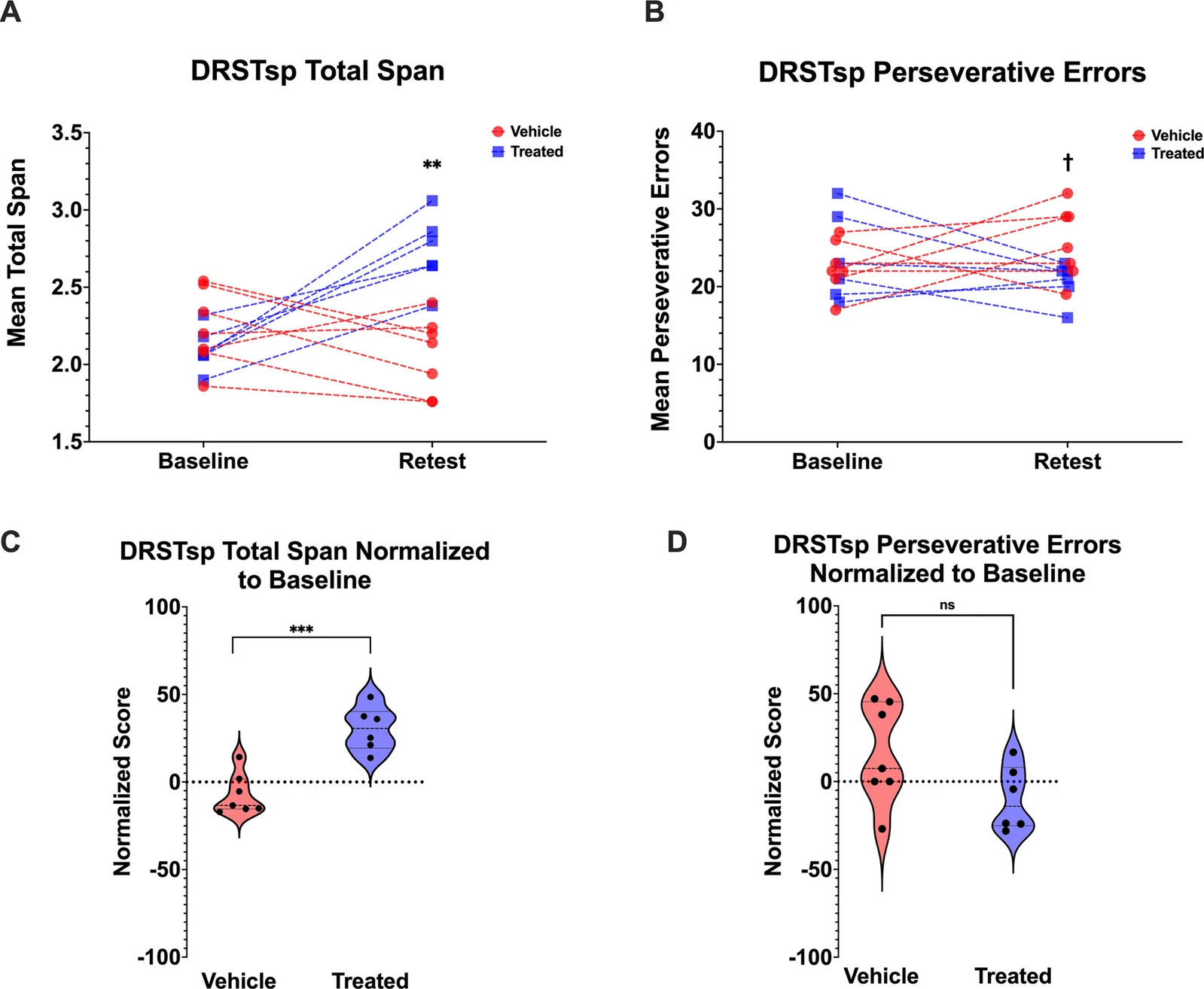
Cognitive Performance on the Delayed Recognition Span Task—Spatial. **A** MSC-EV-treated monkeys achieved significantly higher DRSTsp total span scores at retesting as compared to vehicle monkeys ([*F*(1, 9) = 19.00, *p* = 0.0018]). **B** Vehicle monkeys exhibited more perseverative responses at retesting compared with MSC-EV–treated monkeys; this effect was marginally significant by two-way ANOVA ([*F*(1, 9) = 5.163, *p* = 0.0492]) but did not reach significance in mixed-model analyses. **C** When normalized to baseline, MSC-EV-treated monkeys showed significantly greater improvement in DRSTsp total spans relative to vehicle monkeys ([*F*(1, 9) = 26.89, *p* = 0.0006]). **D** Perseverative errors normalized to baseline did not differ significantly between groups ([*F*(1, 9) = 3.232, *p* = 0.1058]). (***p < 0.001, **p < 0.01, † significant by ANOVA only, not confirmed by mixed model)

Spatial working memory capacity was assessed using DRSTsp total spans, defined as the maximum sequence length reached before error or reaching a maximum length of 9 (Table 3). At baseline, there were no significant effects of treatment group ([*F*(1,9) = 0.8928, *p* = 0.3694]; two-way ANOVA), sex ([*F*(1,9) = 0.4170, *p* = 0.5345]), or group × sex interaction ([*F*(1,9) = 0.7930, *p* = 0.3964]) (Fig. 2A).

#### Delayed Recognition Span Task—Spatial—Perseverative Errors

Perseverative errors on the DRSTsp, defined as repeated responses to the immediately previously selected spatial location, were analyzed as a measure of cognitive flexibility (Table 3). No significant group ([*F*(1,9) = 0.5575, *p* = 0.4743]; two-way ANOVA), sex ([*F*(1,9) = 2.699, *p* = 0.134]), or group × sex interaction effects ([*F*(1,9) = 0.419, *p* = 0.534]) were observed at baseline (Fig. 2B).

Overall, baseline testing revealed limited group or sex differences, except for a female advantage in DNMS acquisition trials and a marginally significant vehicle group advantage in DNMS 2-min delay accuracy.

### Retesting following 18 months of treatment

#### Delayed Non-Matching to Sample—Reacquisition

At retesting, reacquisition on the DNMS task reflects both recognition learning and the capacity to recall and apply the task rule over extended periods. No significant effects of treatment group (trials: [*F*(1,9) = 0.2271, *p* = 0.6450]; errors: [*F*(1,9) = 0.0427, *p* = 0.8408]; two-way ANOVAs) or sex (trials, [*F*(1, 9) = 0.4112, *p* = 0.5373]; errors, [*F*(1, 9) = 0.1595, *p* = 0.6989]) were detected (Table 2). There was also no sex by group interaction for trials ([*F*(1, 9) = 0.579, *p* = 0.466]) or errors ([*F*(1, 9) = 0.525, *p* = 0.487]).

Normalized-to-baseline scores were calculated to assess change in performance from baseline to retesting. For DNMS reacquisition, lower values (negative scores) indicate improvement, reflecting fewer trials or errors required to reach criterion at retesting compared to baseline, whereas higher values (positive scores) indicate a decline in performance. Analysis of these normalized scores revealed no significant effects of group, sex, or their interaction on trials to criterion with two-way ANOVAs (group, [*F*(1, 9) = 0.006216, *p* = 0.9389]; sex, [*F*(1, 9) = 0.08179, *p* = 0.7814]; group × sex, [*F*(1, 9) = 0.807, *p* = 0.392]) or errors to criterion (group, [*F*(1, 9) = 0.05008, *p* = 0.8279]; sex, [*F*(1, 9) = 0.1905, *p* = 0.6728]; group × sex, [*F*(1, 9) = 0.527, *p* = 0.486]) (Table 2).

To determine if there was an effect of testing day or treatment group on DNMS performance over the retesting period, a two-way repeated measures ANOVA was conducted. This analysis revealed no significant effect of testing day ([*F*(2.920, 11.85) = 0.8814, *p* = 0.476]) or group ([*F*(1, 11) = 0.01840, *p* = 0.8945]) as well as no testing day by group interaction ([*F*(17, 69) = 0.9905, *p* = 0.4790]) (Supplementary Fig. 1).

Overall, these results suggest that there was no effect of MSC-EVs on performance on the DNMS basic task, a task of recognition memory, in terms of trials or errors to reach criterion.

#### Delayed Non-Matching to Sample—2- and 10-min Delays

Short- and longer-delay recognition memory were assessed using DNMS 2- and 10-min delay conditions (Table 2). At retesting, MSC-EV-treated monkeys showed significantly greater improvement on 2-min delay performance normalized to baseline (11.25 ± 4.4 vs. −6.17 ± 5.8; [*F*(1,9) = 5.743, *p* = 0.0401]; two-way ANOVA). No significant sex effect was observed ([*F*(1, 9) = 1.036, *p* = 0.3353]; two-way ANOVA). For 10-min delays, there were no significant group ([*F*(1, 9) = 0.008121, *p* = 0.9302]), sex ([*F*(1, 9) = 0.04613, *p* = 0.8347]), or interaction effects ([*F*(1, 9) = 0.0017, *p* = 0.968]; two-way ANOVA).

When comparing performance directly at the retest timepoint, analysis revealed no significant effect of sex ([*F*(1, 9) = 0.1129, *p* = 0.7446]) or group ([*F*(1, 9) = 0.4027, *p* = 0.5415]) on 2-min delays and no effect of sex ([*F*(1, 9) = 0.6021, *p* = 0.4577]) or group ([*F*(1, 9) = 0.3643, *p* = 0.5610]) on 10-min delays.

Because two-way ANOVAs compare group means at single time points, we also conducted mixed-model analyses to account for repeated measures and inter-individual variability across sessions. This approach verifies that group differences were not driven by unequal variances or baseline imbalances and provides a more conservative test of treatment effects. Importantly, the mixed-model results converged with the ANOVA findings, confirming a significant group × time interaction at the 2-min delay (F(1,11) = 5.413, *p* = 0.040).

These results, taken together with the results from the DNMS basic task, suggest that systemic infusions of MSC-EVs biweekly over an 18-month period do not have a significant effect on recognition memory following a relatively short (10 s) or longer (10 min) delay, but interestingly, treatment does influence performance on 2-min delays over time. Mixed-model analyses confirmed this treatment-related effect by detecting a significant group × time interaction, verifying the improvement while accounting for repeated measures. This result is interesting, as there was a baseline group difference on the 2-min delay task, but it was the animals in the vehicle group performing at a higher level. Yet at retesting, not only did the performance by MSC-EV-treated monkeys improve, but the vehicle group’s performance also significantly decreased over time.

#### Delayed Recognition Span Task—Spatial—Total Span

Spatial working memory span was reassessed after treatment with the DRSTsp (Table 3). MSC-EV-treated monkeys demonstrated significantly greater spans at retesting compared to vehicle monkeys (2.73 ± 0.09 vs. 2.06 ± 0.09; [*F*(1,9) = 19.00, *p* = 0.0018]; two-way ANOVA) (Table 3, Fig. 2A). Analyses revealed no significant effect of sex ([*F*(1, 9) = 0.1296, *p* = 0.7272]) and no group by sex interaction ([*F*(1, 9) = 0.2113, *p* = 0.6567]) on DRSTsp total span.

Normalized-to-baseline scores also confirmed a significant treatment effect (30.35 ± 5.2 vs. −7.17 ± 4.4; [*F*(1,9) = 26.89, *p* = 0.0006]; two-way ANOVA) with no effect of sex ([*F*(1, 9) = 0.5372, *p* = 0.4823]) and no group by sex interaction ([*F*(1, 9) = 1.275, *p* = 0.2880]) (Fig. 2C). Mixed-model analyses further showed a robust group × time interaction ([*F*(1,11) = 30.959, *p* < 0.001]), indicating that MSC-EV treatment both maintained and enhanced spatial span capacity over time, while monkeys that received vehicle only either did not improve in performance or declined over time on the DRSTsp, a task of spatial working memory.

Changes in working memory span over time were further examined using linear slope analysis of mean performance from baseline to retesting (Supplementary Fig. 2 A). Treated monkeys exhibited a positive slope (0.63), indicating improved span, whereas vehicle monkeys showed a negative slope (−0.17), consistent with decline; these slopes differed significantly (*p* = 0.0002, Student’s *t*-test). This pattern further suggests that MSC-EV treatment may not only be able to slow down or halt age-related cognitive decline in performance on this task, but in fact potentially reverse typical aging changes in working memory.

Span performance across successive blocks of 5 trials was also evaluated to assess the consistency of treatment effects (Supplementary Fig. 3). Analysis revealed a significant main effect of group ([*F*(1, 11) = 11.40, *p* = 0.0062]; repeated measures ANOVA), with no main effect of trial ([*F*(3.768, 41.44) = 2.216, *p* = 0.0874]) and no group × trial interaction ([*F*(9, 99) = 0.8283, *p* = 0.5917]). Bonferroni-corrected comparisons showed treated monkeys outperformed vehicle monkeys at trial blocks 10 (*p* = 0.0458), 20 (*p* = 0.0136), 40 (*p* = 0.0129) and 45 (*p* = 0.0084). These results demonstrate that MSC-EV-treated monkeys consistently maintained their higher spans across retesting.

Together, these analyses demonstrate a robust treatment-related benefit on spatial working memory span. MSC-EV-treated monkeys not only maintained performance at retesting but also showed significant improvement relative to baseline, whereas vehicle monkeys declined. These effects were consistently supported across group mean comparisons, normalized-to-baseline analyses, mixed-model testing, and slope-based assessments, indicating that MSC-EVs both preserved and enhanced working memory capacity over the 18-month treatment period.

#### Delayed Recognition Span Task—Spatial—Perseverative Errors

Perseverative error frequency was also measured again at retesting. At retesting, MSC-EV-treated monkeys made fewer perseverative errors than vehicle monkeys (20.67 ± 1.0 vs. 25.57 ± 1.7) (Table 3, Fig. 2B). A two-way ANOVA revealed a marginally significant group effect ([*F*(1,9) = 5.163, *p* = 0.0492]; two-way ANOVA), with no significant effect of sex ([*F*(1,9) = 2.803, *p* = 0.1284]) or group × sex interaction ([*F*(1,9) = 0.129, *p* = 0.7280]).

Mixed-model analysis was conducted to account for repeated measures across baseline and retesting. This model did not confirm the ANOVA finding, with no significant main effect of group ([*F*(1,9) = 0.625, *p* = 0.450]; mixed model), no effect of sex ([*F*(1,9) = 5.013, *p* = 0.052]), and no main effect of time ([*F*(1,11) = 0.000, *p* = 1.000]). Importantly, the group × time interaction trended toward significance but did not reach threshold ([*F*(1,11) = 3.877, *p* = 0.075]).

Normalized-to-baseline scores, which quantify change in perseverative errors between baseline and retesting, were also examined (Table 3, Fig. 2D). Here, lower (more negative) values indicate improvement (fewer perseverative errors over time). No significant group differences were observed ([*F*(1,9) = 3.232, *p* = 0.106]; two-way ANOVA) with no effect of sex ([*F*(1, 9) = 0.04773, *p* = 0.8319]) and no group by sex interaction ([*F*(1, 9) = 0.1754, *p* = 0.6852]). However, MSC-EV-treated monkeys showed a trend toward fewer errors over time (–9.75 ± 7.5) compared to vehicle monkeys (+ 15.87 ± 10.6).

We also conducted a linear analysis of change in task performance to further explore the directionality of change in each group on the DRSTsp in terms of perseverative errors (Supplementary Fig. 2B). A linear slope was calculated for each group between baseline and retesting scores for mean perseverative errors, similar to the calculation for mean total span. However, treated (slope = 3.00) and vehicle (slope = −3.00) slopes did not significantly differ (*p* = 0.0747) for perseverative errors when analyzing data in this fashion with a Student’s *t*-test. Notably, the group slopes showed a similar pattern to that observed with total span, again suggesting that treatment improved overall DRSTsp performance.

Together, these analyses suggest that while MSC-EV–treated monkeys tended to exhibit fewer perseverative responses at retesting, the effect was only marginally significant with ANOVA and not consistently supported by mixed-model or normalized analyses, indicating that the treatment-related benefits for perseverative errors are less robust than for span performance.

### MRI markers of brain structural and functional properties

To investigate the effects of MSC-EV treatment on brain structural and functional network changes in vivo, we assessed longitudinal diffusion MRI changes and terminal rs-fMRI outcomes after 18 months of treatment. Two-way ANOVA revealed no significant effect of treatment group on either WM dMRI GFA or RSN measures at the 18-month end of treatment timepoint. No sex or sex × group interaction effects were detected either. However, baseline-normalized dMRI GFA measures were significantly higher in the MSC-EV-treated group compared to the vehicle group in the right middle temporal WM ([*F*(1,9) = 5.825, *p* = 0.039]) (Supplementary Table 1).

**Fig. 3:**
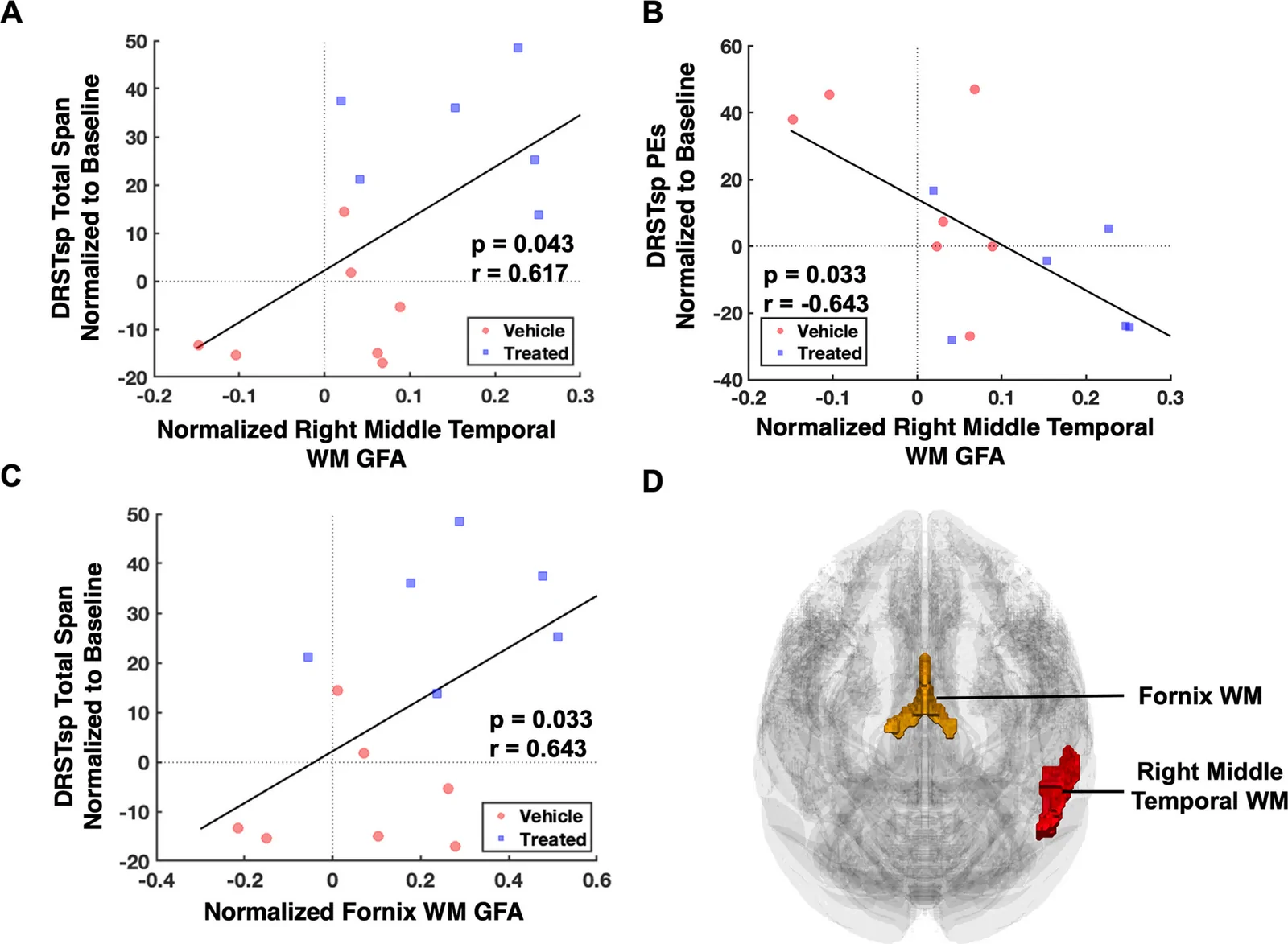
Correlations between Delayed Recognition Span Task—Spatial Performance and dMRI Measures of White Matter Integrity. Increased white matter integrity (normalized rate change of GFA) in the right middle temporal gyrus is correlated with **A** higher baseline-normalized spans on the DRSTsp (*p* = 0.043, *r* = 0.617), and **B** fewer baseline-normalized perseverative errors (PEs) on the DRSTsp (*p* = 0.033, *r* = −0.643). Improved performance on the DRSTsp in terms of total span is also correlated with increased white matter integrity in the **C** fornix (*p* = 0.033, *r* = 0.643). **D** 3D rendering of white matter regions that are correlated with DRSTsp performance (orange: fornix; red: right middle temporal)

We then assessed the relationships between MRI measures of brain structural and functional network integrity with cognitive performance. Correlation analysis revealed significant relationships between the normalized dMRI GFA in the right middle temporal WM and DRSTsp total spans normalized to baseline (*p* = 0.043, *r* = 0.617, fdr *p* = 0.689) (Fig. 3A, D, Supplementary Table 2) as well as with DRSTsp perseverative errors normalized to baseline (*p* = 0.033, *r* = −0.643, fdr *p* = 0.998) (Fig. 3B). While there were no significant group differences in the normalized dMRI GFA in frontal WM, a significant correlational relationship was observed between baseline normalized DRSTsp total spans and the fornix (*p* = 0.033, *r* = 0.643, fdr *p* = 0.689) (Fig. 3C, 3D, Supplementary Table 2).

**Fig. 4:**
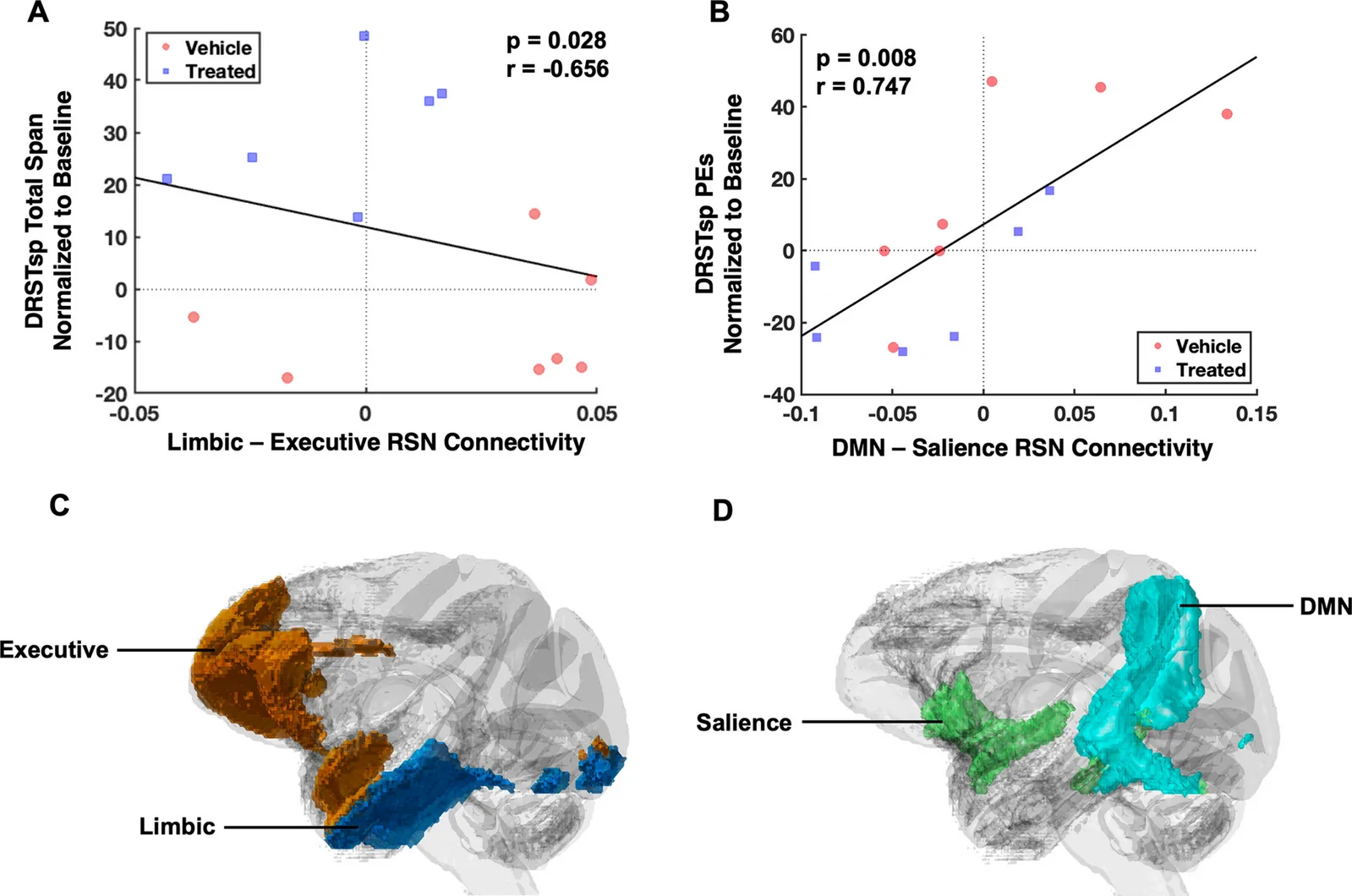
Correlations between Delayed Recognition Span Task—Spatial Performance and Resting-State Network Connectivity. **A** Decreased limbic—executive network functional connectivity at the terminal timepoint is correlated with greater baseline-normalized DRSTsp spans (*p* = 0.028, *r* = −0.656). **B** Decreased Default Mode Network (DMN)—salience network functional connectivity at the terminal time point is correlated with fewer baseline-normalized perseverative errors (PEs) on the DRSTsp (*p* = 0.008, *r* = 0.747). **C** 3D rendering of limbic—executive (blue: limbic network; dark orange: executive network) and **D** DMN—salience (cyan: DMN; green: salience) resting-state functional networks that are correlated with DRSTsp performance measures

Additionally, terminal RSN connectivity between the limbic (consisting of caudal and middle entorhinal, area 35, and area 36 WM) and executive (consisting of area 32, temporal pole, rostral entorhinal, area 9, and ventral area 46 WM) networks was significantly correlated with baseline-normalized DRSTsp total spans (*p* = 0.028, rho = −0.656, fdr *p* = 0.425) (Fig. 4A, C, Supplementary Table 3). A similar significant relationship was also observed between baseline-normalized perseverative errors and the connectivity between the default mode network (DMN; consisting of middle temporal area, ventral area 23, area 7, and medial superior temporal WM) and the salience network (consisting of inferior frontal, rostral temporal, and insular WM) (*p* = 0.008, *r* = 0.747, fdr *p* = 0.124) (Fig. 4B, D, Supplementary Table 3). Together, MRI measures of structural and functional network changes over time in the middle temporal and frontal regions were correlated with longitudinal treatment-related changes in cognitive performance outcomes.

## Discussion

### Summary of results

The current study utilized cognitive testing and diffusion and functional MRI analyses to investigate the effects of MSC-EV treatment on age-related changes in cognition, WM integrity, and brain functional network connectivity in late middle-aged rhesus monkeys. The overall findings of this study showed that MSC-EV treatment: (1) improves spatial working memory performance; (2) shows a trend toward reduced perseverative responses on a spatial working memory task, though this effect was not consistently significant across analyses; and (3) preserves structural and functional states in temporal brain regions over time, which correlated with enhanced working memory performance. Collectively, these results suggest that long-term systemic administration of MSC-EVs in aging rhesus monkeys slows and perhaps reverses age-related cognitive decline on a task of spatial working memory potentially through preservation of WM integrity and functional network connectivity.

### Long-term MSC-EV treatment does not improve recognition memory

The DNMS task is a well-established medial temporal lobe task that measures recognition memory in primates and is sensitive to age-related changes in the brain [22]. Older monkeys are impaired on the DNMS task, requiring substantially more trials and making more errors to learn the non-matching principle compared to younger and middle-aged monkeys [22, 87]. Overall, the current data largely revealed no significant between-group differences on DNMS performance, except for treated monkeys showing a small but significantly greater improvement in DNMS 2-min delay performance (greater normalized score) over time compared to vehicle monkeys. These relatively modest effects of MSC-EV treatment on DNMS are similar to data from our other therapeutic study in rhesus monkeys [88] and are likely related to the fact that the ages of the monkeys in these studies were younger than when we typically observe significant impairments on this task. Previous research has shown that significant deficits on DNMS typically emerge in monkeys over 20–25 years of age [22, 87]. The monkeys in the current study were middle-aged (17–24 years of age), potentially at an age when they only have mild deficits on this task at baseline, and therefore the lack of group difference reflects a “ceiling effect” on their performance. Future studies with older monkeys could determine whether MSC-EVs confer broader benefits to recognition memory in animals with more pronounced deficits in this cognitive domain.

### Long-term MSC-EV treatment improves working memory

An age-related decline in performance on tasks measuring executive function, especially working memory, is well characterized in both humans [16, 17, 89] and non-human primates [2, 8, 9, 18, 21, 34, 90–93]. These early changes are associated with age-related dysfunction of the prefrontal cortex [1, 5, 14, 22, 35, 94–96], driven by region-specific alterations to neuronal spine density, altered firing properties, increased inflammation, and changes in monoamine receptors [29, 97–100]. In addition, age-related impairments in working memory are also linked to significant changes in WM and myelin integrity [8, 36, 101–105].

The DRSTsp, a working memory task employed in the present study, has been shown to be sensitive to these age-related changes in the prefrontal cortex [23, 27, 88]. Here in the present study, monkeys in both the vehicle and treated groups at baseline testing performed below the levels of performance reported in young adult monkeys [23, 27]. However, here we have shown that long-term MSC-EV treatment can shift this age-related trajectory of working memory impairment in middle-aged monkeys. Treated monkeys displayed significantly improved performance on the DRSTsp between baseline and retesting as compared to vehicle monkeys. Specifically, treated monkeys demonstrated significantly higher spans than vehicle monkeys after 18 months of treatment and longer memory spans at retesting compared to their own baseline performance (Fig. 2A, C). This pattern of continuous decline in the vehicle monkeys is what would typically be expected during aging, and the fact that not only did the treated monkeys not decline in performance, but actually improved over time, demonstrates the significant cognitive benefits of MSC-EVs. Notably, monkeys that received MSC-EVs performed at a level equivalent to young adult monkeys measured in one of our previous studies when comparing mean total DRSTsp spans [23]. These striking results indicate that MSC-EV treatment may not only slow age-related declines in working memory but also reverse age-related impairment and improve memory performance in this cohort of middle-aged monkeys.

A particularly interesting finding in this study was that treated monkeys trended toward a decrease in perseveration as compared to vehicle control monkeys. Cognitive perseveration is a response pattern commonly observed in human aging and is characterized by repeating a previously rewarded response strategy for additional trials even when this strategy is not effective nor rewarded [106]. A large body of research has indicated a link between this increased tendency for perseverative responses and age-related changes in prefrontal areas [12, 13, 32, 107–111]. In support of this notion, our group has shown that rhesus monkeys demonstrate an increase in the frequency of perseverative responses that is positively correlated with age beginning in middle age [9, 27]—a deficit that showed improvement with long-term MSC-EV treatment in the present study. At retesting, MSC-EV-treated monkeys made fewer perseverative responses than vehicle monkeys, and this effect reached marginal significance (Fig. 2B, Supplementary Fig. 2B). MSC-EV treatment appears to be trending toward shifting the typical age-related pattern of increased perseveration, evidenced by the positive slope of the vehicle monkeys, toward a negative slope representing a reduction in perseveration over time. It is possible that with increased group sizes, and possibly longer treatment time, this relationship would be significant. Nevertheless, the overall trend is consistent with the more robust treatment-related improvements in spatial working memory over time observed in this cohort, suggesting that MSC-EV treatment may influence multiple aspects of cognitive aging.

Overall, our data reveal that MSC-EVs can slow or ameliorate age-related working memory impairments. While studies in rodent models of Alzheimer’s Disease have shown the potential of MSC-EVs to reduce memory impairments [112, 113], our study is the first to demonstrate their efficacy as a long-term intervention against non-pathological, normal age-related decline in a non-human primate species.

### MSC-EV effects on in vivo brain structural integrity and functional connectivity associated with improved working memory performance

Age-related cognitive impairments in humans have been found to be associated with age-related decreases in diffusion MRI measures (such as GFA) of prefrontal and temporal WM integrity [114–117]. Previous work in rhesus monkeys from our group has similarly shown that, compared to young monkeys, aged monkeys exhibited lower diffusion MRI WM integrity associated with impaired performance on tasks measuring executive function [44]. Our current data revealed that long-term MSC-EV treatment alters age-related WM changes in temporal lobe regions over time, and changes in WM integrity of the fornix and temporal regions are associated with improvements in spatial working memory. Specifically, compared to the vehicle control group, longitudinal MSC-EV treatment prevented age-related decreases in GFA—an estimation of crossing fibers, axonal integrity, and myelination [78]—in right middle temporal gyrus WM over time.

MSC-EV-related improvements in DRSTsp performance were significantly correlated with greater WM integrity in the right middle temporal gyrus [118–124]. Performance on the DRSTsp is primarily mediated by the prefrontal cortex, but specific temporal and parietal regions are also important for spatial working memory [29, 94, 118–120, 122–125]. Notably, the right middle temporal gyrus emerged in our study as a region where WM integrity was preserved with MSC-EV treatment. Although not traditionally emphasized in working memory networks, it serves as an integrative region connecting temporal, parietal, and occipital cortices thereby supporting visuospatial processing and associative memory functions [126–128]. Preservation of WM integrity in this region may facilitate efficient communication between sensory input and higher-order prefrontal systems required for DRSTsp performance. Therefore, the effect of MSC-EV treatment on the integrity of pathways interlinking these different areas may be critical to improved performance on the DRSTsp.

In contrast, the hippocampal formation and parahippocampal cortices, which have well-known roles in memory and spatial navigation [129–134], did not correlate with performance on the DNMS task. Because DNMS performance is thought to rely more heavily on hippocampal processes and tends to be affected later in age, these findings highlight region- and pathway-specific differences in age-related WM pathology which can differentially affect network function and behavior over time. Such differences may explain why MSC-EV treatment influences temporal–parietal–prefrontal circuits more strongly than hippocampal networks. While previous studies in rhesus monkeys have shown WM and myelin pathology in various cortical areas [36, 103, 135, 136], the relationship and temporal structural changes in the WM axons interconnecting distinct regions with age remain largely unknown. Indeed, the hippocampal formation has been previously linked to performance on the DRSTsp in rhesus monkeys [137], which may be related to its connections to the prefrontal cortices [138, 139]. In vivo electrophysiological recordings in monkeys also showed that both hippocampal and prefrontal neurons maintained representations of stimuli over time during an associative memory task [140]. Interestingly, we did observe a significant correlation between DRSTsp performance and WM integrity in the fornix which is the major WM tract projecting from the hippocampal areas and has been shown to be involved in spatial learning tasks in monkeys [141].

Our dMRI data on WM structural integrity suggest that MSC-EV improvements in working memory can result from changes in these cognitive pathway interactions. Age-related changes in WM integrity can result in altered connectivity profiles of distinct functional resting-state networks (RSNs) governed by multiple brain regions [76, 142–144]. Age-related increases in functional connectivity between these RSNs have been reported in both normal aging human lifespan studies and populations with mild cognitive impairments [145, 146]. In our previous study, we reported that greater functional but weaker structural connection strengths of hippocampal to prefrontal cortical networks were associated with impairments in DRSTsp performance in middle-aged rhesus monkeys [76], suggesting a complex relationship between age-related WM damage and compensatory strengthening of correlated network activity, specifically in frontal and temporal regions, that can underlie age-related cognitive decline [45, 76, 147, 148]. In the current study, we report increased structural integrity in dMRI middle temporal lobe WM GFA following 18 months of MSC-EV treatment, accompanied by weaker functional connectivity between the default mode network and the salience network as well as the limbic and executive network, involving connections between inferior frontal, rostral temporal, and insular WM areas, and entorhinal and frontotemporal areas, respectively. This suggests that MSC-EV treatment appears to ameliorate age-related structural–functional changes in brain networks by preserving WM integrity and concomitantly recovering between-network functional connectivity to a state more similar to young brains which may underlie improvements in spatial working memory.

### Conclusions and future directions

Overall, this study demonstrates that long-term systemic MSC-EV treatment in middle-aged rhesus monkeys mitigates age-related cognitive decline and improves in vivo measures of WM structure and network function. Specifically, MSC-EV treatment improved spatial working memory performance and reduced perseverative responses. These cognitive improvements were correlated with preservation of temporal WM structural integrity and normalization of large-scale functional brain network connectivity in prefrontal and temporal regions. In our current data, due to the imbalance between the sample size and the imaging features, most comparisons did not reach statistical significance after FDR correction. Additionally, while sex and sex interaction effects were tested in this study, we did not observe widespread brain MRI differences based on sex effects. A larger sample size with a longer treatment period for future studies may improve statistical power. From our longitudinal dMRI findings, which reflect overall changes in myelination and axonal integrity, we did not observe widespread group differences between treatment groups. This suggests that more subtle molecular changes undetectable by MRI, potentially due to the relatively young age of these monkeys and absence of large-scale WM degeneration, may be present and warrant further investigation. Anatomical tract-tracing and histological analysis of prefrontal and medial temporal lobe WM pathways will also be necessary to further explore the treatment-related improvement in WM integrity and network connectivity shown here.

Nevertheless, our findings suggest that MSC-EVs can mitigate or even reverse early age-related cognitive decline by preserving brain structure and function related to working memory. Importantly, this study highlights the potential of MSC-EVs as a viable, minimally invasive therapeutic approach for promoting healthy cognitive aging in higher-order primates, with implications for translational development in aging human populations.

## Supplementary Information

Below is the link to the electronic supplementary material.

ESM 1 (DOCX 9.91 MB)

## Data Availability

The data presented in this study are available from the corresponding author upon reasonable request.
