# Supplementary material for: Mesenchymal cell-derived extracellular vesicles ameliorate age-related deficits in working memory and in vivo MRI measures of white matter structure and function in rhesus monkeys
Source: GeroScience. 2025 Dec 2;48(5):6775–99. doi: 10.1007/s11357-025-01992-0 (PMC13601405; doi:10.1007/s11357-025-01992-0)
Supplement: Supplementary file 1 [file 11357_2025_1992_MOESM1_ESM.docx]

**Supplementary Table 1.**

Group comparisons of dMRI White Matter GFA normalized rate change.

|  | **Treatment effect** | | **Sex effect** | | **Interaction effect** | |
| --- | --- | --- | --- | --- | --- | --- |
|  | **p** | **F(1,9)** | **p** | **F(1,9)** | **p** | **F(1,9)** |
| Genu of Corpus Callosum | 0.853 | 0.037 | 0.353 | 0.959 | 0.682 | 0.179 |
| Body of Corpus Callosum | 0.791 | 0.075 | 0.310 | 1.157 | 0.443 | 0.643 |
| Splenium of Corpus Callosum | 0.924 | 0.010 | 0.204 | 1.879 | 0.755 | 0.103 |
| Fornix | 0.108 | 3.178 | 0.050 | 5.102 | 0.368 | 0.900 |
| Corticospinal Tract - Right | 0.941 | 0.006 | 0.851 | 0.037 | 0.628 | 0.251 |
| Corticospinal Tract - Left | 0.744 | 0.113 | 0.276 | 1.344 | 0.609 | 0.281 |
| Anterior Limb of the Internal Capsule - Right | 0.751 | 0.107 | 0.237 | 1.603 | 0.651 | 0.219 |
| Anterior Limb of the Internal Capsule - Left | 0.957 | 0.003 | 0.516 | 0.457 | 0.673 | 0.190 |
| Posterior Limb of the Internal Capsule - Right | 0.934 | 0.007 | 0.813 | 0.060 | 0.476 | 0.553 |
| Posterior Limb of the Internal Capsule - Left | 0.505 | 0.481 | 0.637 | 0.238 | 0.312 | 1.149 |
| Retrolenticular Limb of the Internal Capsule - Right | 0.771 | 0.090 | 0.712 | 0.145 | 0.513 | 0.465 |
| Retrolenticular Limb of the Internal Capsule - Left | 0.456 | 0.606 | 0.728 | 0.129 | 0.302 | 1.198 |
| Anterior Corona Radiata - Right | 0.887 | 0.021 | 0.655 | 0.213 | 0.443 | 0.645 |
| Anterior Corona Radiata - Left | 0.503 | 0.486 | 0.489 | 0.520 | 0.827 | 0.051 |
| Superior Corona Radiata - Right | 0.976 | 0.001 | 0.652 | 0.217 | 0.483 | 0.536 |
| Superior Corona Radiata - Left | 0.744 | 0.113 | 0.954 | 0.003 | 0.701 | 0.157 |
| Posterior Corona Radiata - Right | 0.533 | 0.421 | 0.466 | 0.579 | 0.482 | 0.537 |
| Posterior Corona Radiata - Left | 0.448 | 0.630 | 0.336 | 1.032 | 0.821 | 0.054 |
| Posterior Thalamic Radiation - Right | 0.422 | 0.708 | 0.311 | 1.155 | 0.838 | 0.044 |
| Posterior Thalamic Radiation - Left | 0.718 | 0.139 | 0.390 | 0.815 | 0.859 | 0.033 |
| Sagittal Striatum - Right | 0.670 | 0.194 | 0.998 | 0.000 | 0.777 | 0.085 |
| Sagittal Striatum - Left | 0.548 | 0.390 | 0.220 | 1.736 | 0.864 | 0.031 |
| External Capsule - Right | 0.526 | 0.436 | 0.476 | 0.554 | 0.715 | 0.142 |
| External Capsule - Left | 0.507 | 0.477 | 0.199 | 1.921 | 0.868 | 0.029 |
| Superior Cingulum - Right | 0.970 | 0.001 | 0.743 | 0.114 | 0.556 | 0.373 |
| Superior Cingulum - Left | 0.355 | 0.949 | 0.764 | 0.096 | 0.795 | 0.071 |
| Perihippocampal Cingulum - Right | 0.895 | 0.018 | 0.431 | 0.680 | 0.529 | 0.429 |
| Perihippocampal Cingulum - Left | 0.233 | 1.636 | 0.313 | 1.144 | 0.794 | 0.072 |
| Stria Terminalus - Right | 0.636 | 0.239 | 0.561 | 0.365 | 0.707 | 0.150 |
| Stria Terminalus - Left | 0.480 | 0.544 | 0.208 | 1.842 | 0.993 | <0.001 |
| Superior Longitudinal Fasciculus - Right | 0.444 | 0.640 | 0.834 | 0.047 | 0.458 | 0.602 |
| Superior Longitudinal Fasciculus - Left | 0.325 | 1.083 | 0.238 | 1.599 | 0.446 | 0.635 |
| Superior Fronto-Occipital Fasciculus - Right | 0.649 | 0.222 | 0.378 | 0.859 | 0.229 | 1.669 |
| Superior Fronto-Occipital Fasciculus - Left | 0.651 | 0.219 | 0.778 | 0.084 | 0.708 | 0.149 |
| Uncinate Fasciculus - Right | 0.164 | 2.290 | 0.261 | 1.441 | 0.409 | 0.748 |
| Uncinate Fasciculus - Left | 0.546 | 0.393 | 0.175 | 2.165 | 0.366 | 0.906 |
| Tapetum - Right | 0.628 | 0.251 | 0.597 | 0.300 | 0.778 | 0.084 |
| Tapetum - Left | 0.967 | 0.002 | 0.072 | 4.151 | 0.880 | 0.024 |
| Anterior Commissure | 0.702 | 0.156 | 0.063 | 4.478 | 0.584 | 0.323 |
| Dorsal Prefrontal WM - Right | 0.976 | 0.001 | 0.226 | 1.687 | 0.631 | 0.247 |
| Dorsal Prefrontal WM - Left | 0.616 | 0.270 | 0.667 | 0.198 | 0.664 | 0.202 |
| Ventral Prefrontal WM - Right | 0.877 | 0.025 | 0.272 | 1.371 | 0.617 | 0.269 |
| Ventral Prefrontal WM - Left | 0.337 | 1.028 | 0.153 | 2.434 | 0.485 | 0.531 |
| Dorsal Posterior Corona Radiata - Right | 0.366 | 0.908 | 0.336 | 1.035 | 0.788 | 0.077 |
| Dorsal Posterior Corona Radiata - Left | 0.510 | 0.472 | 0.485 | 0.529 | 0.858 | 0.034 |
| Inferior Frontal Gyrus WM - Right | 0.108 | 3.186 | **0.013** | 9.638 | 0.259 | 1.455 |
| Inferior Frontal Gyrus WM - Left | 0.784 | 0.080 | 0.145 | 2.540 | 0.274 | 1.355 |
| Superior Temporal Gyrus WM - Right | 0.928 | 0.009 | 0.653 | 0.216 | 0.472 | 0.564 |
| Superior Temporal Gyrus WM - Left | 0.121 | 2.937 | **0.050** | 5.145 | 0.172 | 2.201 |
| Middle Temporal Gyrus WM - Right | **0.039** | 5.825 | 0.318 | 1.116 | 0.654 | 0.215 |
| Middle Temporal Gyrus WM - Left | 0.437 | 0.663 | **0.045** | 5.442 | 0.437 | 0.663 |
| Adjacent Thalamus WM - Right | 0.830 | 0.049 | 0.496 | 0.503 | 0.866 | 0.030 |
| Adjacent Thalamus WM - Left | 0.997 | <0.001 | 0.937 | 0.007 | 0.565 | 0.357 |
| Adjacent Amygdala White Matter - Right | 0.611 | 0.277 | 0.770 | 0.091 | 0.511 | 0.469 |
| Adjacent Amygdala White Matter - Left | 0.951 | 0.004 | 0.887 | 0.021 | 0.480 | 0.543 |
| Anterior Cingulum WM - Right | 0.289 | 1.272 | 0.410 | 0.747 | 0.832 | 0.048 |
| Anterior Cingulum WM - Left | 0.987 | <0.001 | 0.095 | 3.482 | 0.256 | 1.472 |

**Supplementary Table 2.**

Correlation analyses between dMRI White Matter GFA normalized rate change and DRSTsp scores normalized to baseline.

|  | **DRSTsp Total Span Normalized to Baseline** | | | **DRSTsp PEs Normalized to Baseline** | | |
| --- | --- | --- | --- | --- | --- | --- |
|  | **p** | **rho** | **fdr p** | **p** | **rho** | **fdr p** |
| Genu of Corpus Callosum | 0.524 | 0.216 | 0.766 | 0.823 | -0.076 | 0.998 |
| Body of Corpus Callosum | 0.348 | 0.313 | 0.689 | 0.551 | -0.202 | 0.998 |
| Splenium of Corpus Callosum | 0.626 | 0.166 | 0.797 | 0.789 | 0.091 | 0.998 |
| Fornix | **0.033** | 0.643 | 0.689 | 0.365 | -0.303 | 0.998 |
| Corticospinal Tract - Right | 0.755 | 0.107 | 0.797 | 0.704 | 0.130 | 0.998 |
| Corticospinal Tract - Left | 0.605 | -0.176 | 0.797 | 0.089 | -0.536 | 0.998 |
| Anterior Limb of the Internal Capsule - Right | 0.753 | 0.107 | 0.797 | 0.882 | -0.051 | 0.998 |
| Anterior Limb of the Internal Capsule - Left | 0.387 | 0.290 | 0.689 | 0.757 | -0.106 | 0.998 |
| Posterior Limb of the Internal Capsule - Right | 0.450 | 0.254 | 0.739 | 0.894 | -0.046 | 0.998 |
| Posterior Limb of the Internal Capsule - Left | 0.700 | 0.132 | 0.797 | 0.594 | -0.181 | 0.998 |
| Retrolenticular Limb of the Internal Capsule - Right | 0.538 | 0.208 | 0.767 | 0.634 | 0.162 | 0.998 |
| Retrolenticular Limb of the Internal Capsule - Left | 0.751 | 0.108 | 0.797 | 0.476 | -0.241 | 0.998 |
| Anterior Corona Radiata - Right | 0.292 | 0.349 | 0.689 | 0.998 | 0.001 | 0.998 |
| Anterior Corona Radiata - Left | 0.197 | 0.421 | 0.689 | 0.565 | -0.195 | 0.998 |
| Superior Corona Radiata - Right | 0.381 | 0.293 | 0.689 | 0.847 | -0.066 | 0.998 |
| Superior Corona Radiata - Left | 0.462 | 0.248 | 0.739 | 0.691 | -0.136 | 0.998 |
| Posterior Corona Radiata - Right | 0.150 | 0.465 | 0.689 | 0.595 | -0.181 | 0.998 |
| Posterior Corona Radiata - Left | 0.294 | 0.348 | 0.689 | 0.363 | -0.304 | 0.998 |
| Posterior Thalamic Radiation - Right | 0.268 | 0.366 | 0.689 | 0.407 | -0.278 | 0.998 |
| Posterior Thalamic Radiation - Left | 0.638 | 0.160 | 0.797 | 0.692 | -0.135 | 0.998 |
| Sagittal Striatum - Right | 0.264 | 0.369 | 0.689 | 0.744 | -0.112 | 0.998 |
| Sagittal Striatum - Left | 0.365 | -0.303 | 0.689 | 0.998 | -0.001 | 0.998 |
| External Capsule - Right | 0.224 | 0.399 | 0.689 | 0.649 | -0.155 | 0.998 |
| External Capsule - Left | 0.217 | 0.405 | 0.689 | 0.517 | -0.219 | 0.998 |
| Superior Cingulum - Right | 0.306 | 0.340 | 0.689 | 0.928 | 0.031 | 0.998 |
| Superior Cingulum - Left | 0.143 | 0.472 | 0.689 | 0.390 | -0.288 | 0.998 |
| Perihippocampal Cingulum - Right | 0.746 | 0.110 | 0.797 | 0.236 | -0.390 | 0.998 |
| Perihippocampal Cingulum - Left | 0.189 | 0.428 | 0.689 | 0.682 | -0.140 | 0.998 |
| Stria Terminalus - Right | 0.997 | 0.001 | 0.997 | 0.967 | -0.014 | 0.998 |
| Stria Terminalus - Left | 0.403 | 0.281 | 0.697 | 0.465 | -0.247 | 0.998 |
| Superior Longitudinal Fasciculus - Right | 0.138 | 0.477 | 0.689 | 0.448 | -0.255 | 0.998 |
| Superior Longitudinal Fasciculus - Left | 0.146 | 0.469 | 0.689 | 0.316 | -0.334 | 0.998 |
| Superior Fronto-Occipital Fasciculus - Right | 0.146 | 0.468 | 0.689 | 0.847 | 0.066 | 0.998 |
| Superior Fronto-Occipital Fasciculus - Left | 0.692 | 0.135 | 0.797 | 0.634 | -0.162 | 0.998 |
| Uncinate Fasciculus - Right | 0.379 | 0.295 | 0.689 | 0.154 | -0.460 | 0.998 |
| Uncinate Fasciculus - Left | 0.498 | 0.229 | 0.747 | 0.164 | -0.451 | 0.998 |
| Tapetum - Right | 0.345 | 0.315 | 0.689 | 0.528 | -0.214 | 0.998 |
| Tapetum - Left | 0.849 | -0.065 | 0.880 | 0.477 | 0.240 | 0.998 |
| Anterior Commissure | 0.562 | 0.197 | 0.781 | 0.356 | -0.308 | 0.998 |
| Dorsal Prefrontal WM - Right | 0.725 | 0.120 | 0.797 | 0.784 | -0.094 | 0.998 |
| Dorsal Prefrontal WM - Left | 0.252 | 0.378 | 0.689 | 0.724 | -0.121 | 0.998 |
| Ventral Prefrontal WM - Right | 0.467 | 0.245 | 0.739 | 0.708 | -0.128 | 0.998 |
| Ventral Prefrontal WM - Left | 0.109 | 0.510 | 0.689 | 0.346 | -0.315 | 0.998 |
| Dorsal Posterior Corona Radiata - Right | 0.173 | 0.442 | 0.689 | 0.326 | -0.327 | 0.998 |
| Dorsal Posterior Corona Radiata - Left | 0.317 | 0.333 | 0.689 | 0.292 | -0.350 | 0.998 |
| Inferior Frontal Gyrus WM - Right | 0.122 | 0.494 | 0.689 | 0.406 | -0.279 | 0.998 |
| Inferior Frontal Gyrus WM - Left | 0.188 | 0.429 | 0.689 | 0.680 | -0.141 | 0.998 |
| Superior Temporal Gyrus WM - Right | 0.491 | 0.233 | 0.747 | 0.985 | 0.006 | 0.998 |
| Superior Temporal Gyrus WM - Left | 0.384 | 0.292 | 0.689 | 0.261 | -0.371 | 0.998 |
| Middle Temporal Gyrus WM - Right | **0.043** | 0.617 | 0.689 | **0.033** | -0.643 | 0.998 |
| Middle Temporal Gyrus WM - Left | 0.087 | 0.540 | 0.689 | 0.890 | -0.047 | 0.998 |
| Adjacent Thalamus WM - Right | 0.963 | -0.016 | 0.981 | 0.412 | -0.276 | 0.998 |
| Adjacent Thalamus WM - Left | 0.717 | -0.124 | 0.797 | 0.863 | 0.059 | 0.998 |
| Adjacent Amygdala White Matter - Right | 0.646 | 0.156 | 0.797 | 0.957 | -0.019 | 0.998 |
| Adjacent Amygdala White Matter - Left | 0.618 | -0.170 | 0.797 | 0.777 | 0.097 | 0.998 |
| Anterior Cingulum WM - Right | 0.310 | 0.338 | 0.689 | 0.406 | -0.279 | 0.998 |
| Anterior Cingulum WM - Left | 0.329 | 0.325 | 0.689 | 0.975 | -0.011 | 0.998 |

**Supplementary Table 3.**

Correlation analyses between terminal RSN measures and DRSTsp scores normalized to baseline.

|  | **DRSTsp Total Span Normalized to Baseline** | | | **DRSTsp PEs Normalized to Baseline** | | |
| --- | --- | --- | --- | --- | --- | --- |
|  | **p** | **rho** | **fdr p** | **p** | **rho** | **fdr p** |
| **Limbic** | 0.747 | 0.110 | 0.920 | 0.631 | 0.164 | 0.728 |
| **Default Mode (DMN)** | 0.634 | -0.162 | 0.920 | 0.396 | 0.285 | 0.722 |
| **Salience** | 0.259 | 0.373 | 0.777 | 0.316 | -0.334 | 0.722 |
| **Control** | 0.491 | 0.233 | 0.920 | 0.493 | 0.232 | 0.722 |
| **Executive** | 0.920 | 0.034 | 0.920 | 0.316 | 0.334 | 0.722 |
| **Limbic - DMN** | 0.316 | 0.334 | 0.790 | 0.415 | 0.274 | 0.722 |
| **Limbic - Salience** | 0.776 | 0.097 | 0.920 | 0.448 | 0.256 | 0.722 |
| **Limbic - Control** | 0.751 | 0.108 | 0.920 | 0.586 | -0.185 | 0.728 |
| **Limbic - Executive** | **0.028** | -0.656 | 0.425 | 0.065 | 0.573 | 0.327 |
| **DMN - Salience** | 0.508 | -0.224 | 0.920 | **0.008** | 0.747 | 0.124 |
| **DMN - Control** | 0.134 | -0.481 | 0.671 | 0.130 | 0.486 | 0.486 |
| **DMN - Executive** | 0.887 | -0.049 | 0.920 | 0.762 | -0.103 | 0.768 |
| **Salience - Control** | 0.916 | -0.036 | 0.920 | 0.768 | 0.101 | 0.768 |
| **Salience - Executive** | 0.226 | -0.397 | 0.777 | 0.530 | 0.213 | 0.722 |
| **Control - Executive** | 0.122 | -0.495 | 0.671 | 0.056 | 0.590 | 0.327 |

**Supplementary Fig. 1**

**Supplementary Fig. 2**

**Supplementary Fig. 3**
